# Relating antennal sensilla diversity and possible species behaviour in the planthopper pest *Lycorma delicatula* (Hemiptera: Fulgoromorpha: Fulgoridae)

**DOI:** 10.1371/journal.pone.0194995

**Published:** 2018-03-27

**Authors:** Rong-Rong Wang, Jia-Jia Liu, Xin-Yu Li, Ai-Ping Liang, Thierry Bourgoin

**Affiliations:** 1 Institute of Zoology, Chinese Academy of Sciences, Chaoyang District, Beijing, China; 2 School of Biological Science and Medical Engineering, Haidian District, Beihang University, Beijing, China; 3 School of Nature Conservation, Beijing Forestry University, Beijing, China; 4 Institut Systématique, Evolution, Biodiversité (ISYEB), UMR 7205 MNHN-CNRS-SorbonneUniversité-EPHE, Museum National d'Histoire Naturelle, CP50, Paris, France; University of Arizona, UNITED STATES

## Abstract

Antennal sensory units in nymphs and adults of the spotted Lanternfly, *Lycorma delicatula* (White) (Hemiptera: Fulgoromorpha: Fulgoridae), an economically important plant pest, are studied with scanning electron microscopy. Sensilla trichodea / chaetica type recognition is based on their external morphology and ratio of their size to diameter. The flagellum Bourgoin’s organ is a complex sensory unit with 2–3 internal sensilla coeloconica. During nymphal stages, the sensory surface available for a chemoreceptive function particularly increases with the number and size of sensilla placodea on the antennal pedicel. From first to fourth instar and to adult males and females, plate organ sensory surface is estimated to increase respectively by 33x, 68x and 125x (= 2.72 mm^2^ and 5.02 mm^2^ respectively for males and females). The most important increase (5x) occurs between second and third instar. In parallel, a distinctive pair of plate organs on the flagellum decreases in size from first to third instar, and disappears. Sexual dimorphism occurs in sensilla placodea in adults. Diversity, disparity and evolution of nymphal sensilla, and their sexual dimorphism in adults are discussed in the context of the species and planthopper behaviour.

## Introduction

The Spotted Lanternfly *Lycorma delicatula* (White) (Hemiptera: Fulgoromorpha: Fulgoridae) is a planthopper used in traditional Chinese medicine since 1100 [1]. In the recent years, it has become an economically important agricultural (esp. grape) and forestry pest (esp. *Ailanthus*), phloem feeding on the phloem of a wide range of host plants [2–4] and causing serious wilting, even death, of those host plants.

*L*. *delicatula* was originally described from China, India (Assam, Bangladesh) and Japan [5], but it has now been considered as introduced to Korea [6] and Vietnam [7]. Economic damage by the species in these countries is increasing annually, especially in Korea [1, 2, 8, 9]. In 2014, it was first recorded from eastern Pennsylvania, USA [4], where it was collected in large numbers (but not only) on its Korean preferred host plant, *Ailanthus altissima* (Mill.) Swingle (Sapindales, Simaroubaceae). Since, it has now spread to the neighbouring states of Delaware, Maryland, New York and in early January 2018 in Virginia [10].

In spite of its pest status, very little is known about *L*. *delicatula* biology [11], which life history and behaviour was recently reviewed [4]. Females lay egg-masses containing 30–50 eggs on tree trunks and other surfaces. There are 4 nymphal instars. Nymphs and adults crawl and jump, but adults are reported as poor flyers [3]. *L*. *delicatula* is univoltine, overwintering as eggs [2]. Instars 1 through 3 may feed upon many different plant species but the fourth instar strongly prefers *Ailanthus* [12]. Intraspecific competition between nymphs [13] and also adult females for feeding sites occurs (E. Smyers pers. comm.) and males aggregate around females (T. Bourgoin, pers. obs.); warning posture in adults with spread of bright warning pattern of hind wings is also reported [14]. Acoustic communication (substrate-borne vibrations on the host plant for short range communication) has not yet been recorded although this is probable as it occurs in all Hemiptera studied [15]. No information is available about dispersal behaviour and its determinants [16].

The insect head capsule use to carry many sensilla with different sensory functions including smell, taste, sound, touch, vision, proprioception, and geo-, thermo-, and hygroreception, that play important roles in the insect behaviour [17,18]. Particularly in obligate phytophagous insects such as Fulgoromorpha, sensory structures regulate interactions (host location, feeding) with host plants. However, in planthoppers, host plants are not only used as sources of food but also as mating, oviposition and shelters sites, and as sites to communicate and disseminate acoustic signals [19–20]. To interact with their host plants, planthoppers possess a wide range of sensilla types that are mostly located on the antenna, particularly large olfactory [21–23] placoid sensilla [24–26] and on the labium [27]. Functional specializations in the distribution of these structures were hypothesized for planthoppers, with the olfactory role mainly devoted to the antennae [23], the gustatory function transferred from antenna to the apex of the labium (planthoppers do not antennate the plant during surface exploration of plant tissues), and the chemo-reception contact function also concentrated at the apex of the labium [27, 28]. These sensilla have evolved with specialized cuticular structures: many are aporous or with a variable number of pores, sometimes organized in rows, with ridges and grooves in various patterns, and differing in both size and shape. They are often difficult to clearly distinguish and, therefore, difficult to evaluate [29].

Little information exists on the cephalic sensory equipment of planthoppers and almost nothing is known about immature stages even though they occupy the longest period of the insect life cycle and usually have the greatest impact on plants. Accordingly, a more accurate knowledge of cephalic sensilla diversity, distribution, and evolution during the different development phases of *L*. *delicatula* might provide a better understanding of dispersal behaviour and its determinism, and insight into possible pest management strategies. The present paper provides therefore the first description of the external morphology, number, and distribution of head sensilla in all stages of *L*. *delicatula*, aiming to better understand the development of these sensory structures, and to relate these results with possible sensory and ecological behaviour of this pest and of planthoppers in general.

## Material and methods

### Sampling

All specimens of *L*. *delicatula* were collected in Beijing Forest University campus and in the city of Beijing. No specific permissions are required to collect freely specimens of this species in China, which is neither an endangered nor a protected species. All first to third instars and most fourth instars were collected on *Parthenocissus quinquefolia* (L.) Planch (Vitaceae) from late-April to late-June. A few fourth instars were also caught on *A*. *altissima* in late-June to mid-July and adults in October.

### Scanning electron microscopy (SEM)

We examined 11 first instars (N1), 8 second instars (N2), 10 third instars (N3), 10 fourth instars (N4), and 6 female and 6 male adults. After their collect, specimens were preserved in phosphate buffered 2.5% glutaraldehyde at 0–4°C for 24 hours. Each specimen was then cleaned by a series of pulses of 10s in a dimethylbenzene bath in an ultrasonic cleaner for 10 min to remove the cuticular waxy powder, and cleaned twice, 5 min for each, in 50% and 75% alcohol. Samples were then dehydrated in a series of graded ethanol concentrations. Entire heads and antennae were mounted on stubs with double-sided adhesive tape, left in a desiccator overnight to dry thoroughly, and then sputter coated with a 60–80 μm gold film. Observations were made with a Hitachi S34Q scanning electron microscope (Hitachi Corp., Tokyo, Japan) at the Microscopy Core Facility, Biological Technology Centre, Beijing Forestry University (Beijing, China). All measurements were done directly on screen or on scaled photos.

### Terminology

Sensilla chaetica have a cuticular shaft thicker than those in sensilla trichodea and the former is not freely moveable [29]. Both are hair-like and might have a smooth or ridged shaft. These characteristics make difficult to clearly distinguish the two types by SEM only. Accordingly, we refer to both of these as sensilla chaetica. Subtypes of sensilla chaetica are usually separated by their length [27]. They were however better and non-ambiguously discriminate using the ratio of their length to their basal diameter (L/BD), allowing a clearer distinction between subtypes by discontinuous values. According to Shields [29] both might be mecano- or contact chemoreceptive but only multiporous sensilla trichodea have an olfactory function. Sensilla terminology is therefore adapted from Shields [29] and Brozek & Bourgoin [27] with their function according to Fu et al. [24]; their morphological characteristics and measurements are summarised in Table 1. A new typology is proposed for plate organs in the discussion part. The four antennal faces are recognized according to the antenna insertion in the antennal socket and we thus recognize a dorsal and ventral face, an anterior face (facing the gena) and a posterior one.

**Table 1:** Terminology used with probable functions, morphological characteristics and measurements, and distribution of antennal sensilla in *Lycorma delicatula*.

Sensilla type	Function	Sensillum morphological characteristics	Measurements	Distribution
Ch1	Chemo-mechanoreceptive sensillum	Robust medium size hair-like sensillum. Socket base wide, slightly elevated after a circular sulcus. Basally straight, then distinctly bent at mid length, blunt-tipped, Shaft not ridged but very slightly rough. No pore visible. Probably a true sensillum trichodeum	L = 20–35.5 μm Ø = 3.5–6.6 μm L/BD = 4.5–6.6	On pecicel and flagellum, at all stages
Ch2	Chemo-mechanoreceptive sensillum	Slender hair-like sensillum chaeticum. Long, straight faintly curved apically. Base regular and acute tipped. Socket base elevated over the body wall, separated from shaft by a circular furrow. Shaft longitudinally ridged, 6–12 ridges visible at base in lateral view, often convergent apically. Several pores visibles.	L = 38–56 μm Ø = 2.45–3.1 μm L/BD = 15.6–19.9	On N2 flagellum base and pedicel at all stages and adults
Ch2L	Chemo-mechanoreceptive sensillum	Same as CH2M but longer sensillum chaeticum. Shaft longitudinally ridged, 9–11 ridges visible at base in lateral view. Several pores visibles.	L = 75–78 μm Ø = 4.2 μm L/BD = 17.8–18.6	On pedicel in N4 and adults
Ch3	Mechanoreceptive sensillum	Medium size hair-like sensillum. Long and straight then slightly bent apically. Acute-tipped. A basal pore present. Shaft ridged, ridges more numerous than in CH2 at base in lateral view, parallel. Probably a sensillum chaeticum	L = 20–32 μm Ø = 2.2–3.3 μm L/BD = 9.5–9.6	On ventrolateral side of scape in all stages
PO1	Chemoreceptive sensillum	Multiporous sensillum placodeum of lobulated ridged type. Surelevated over body wall with basal furrow clearly visible. External non-porous cuticular denticules short, basally conical, with a smooth surface	Ø = 12–70 μm	On flagellum basal bulb from N1 to N3
PO2	Chemoreceptive sensillum	Multiporous sensillum placodeum of lobulated ridged type. At the level of body wall, basal furrow hidden below body wall, weekly visible. External non-porous cuticular denticules robust, basally triangular, longitudinally widely sulcated	Ø = 37.8–80 μm	On pedicel from N2 to adults
CA1	Proprioceptive sensillum	Dome-shaped aporous sensillum campaniformium. Limited by an irregular circular ridge	Ø = 12.49–18.25 μm	One on pedicel apex and one on scape
CA2	Proprioceptive sensillum	Dome-shaped aporous sensillum campaniformium. A central cupule. Limited by an irregular circular ridge	Ø = 9.99–11.5 μm	In N4 and adults on posterior face of pedicel
CO	Thermo-, hygro- and/or chemoreceptive sensillum	Elevated dome-shaped porous sensillum coeloconicum. A central pore at the top of a strongly elevated and ridged dome		Inside Bourgoin's organ

BD: sensillum basal diameter range; L: sensillum length range; Ø: sensillum basal diameter range.

### Plate organs sensory surface calculation

In a simple approximation, a sensory plate organ in *L*. *delicatula* can be described as a rounded disc bearing a series of smaller rounded and free perpendicular disc-like lobes. Accordingly, if ∅PO and ∅Lob are respectively the diameter of a plate organ and a lobe, and NbLob, the number of lobes carried by a plate organ, we approximated the sensory surface (SS) occupied by each unit using the sum of the surface of the plate organ assimilated to a disc [(∅PO/2)^2^×π], plus the sensory surface of a lobe-like expansion approximated to a double-faced disc [2×((∅Lob/2)^2^×π)], by the number of lobes per plate organ, according the following formula:

$$\mathrm{SS}=({\left(\emptyset \mathrm{PO}/2\right)}^{2}\times \pi )+\left((2\times ({\left(\emptyset \mathrm{Lob}/2\right)}^{2}\times \pi ))\times \mathrm{NbLob}\right)$$ (1)

All measurements are given in μm, Means for measurement (sensilla chaetica) and for numbers of sensilla placodea were used for the synthetic results presented in S1 and S2 Appendixes.

## Results

**Fig 1:**
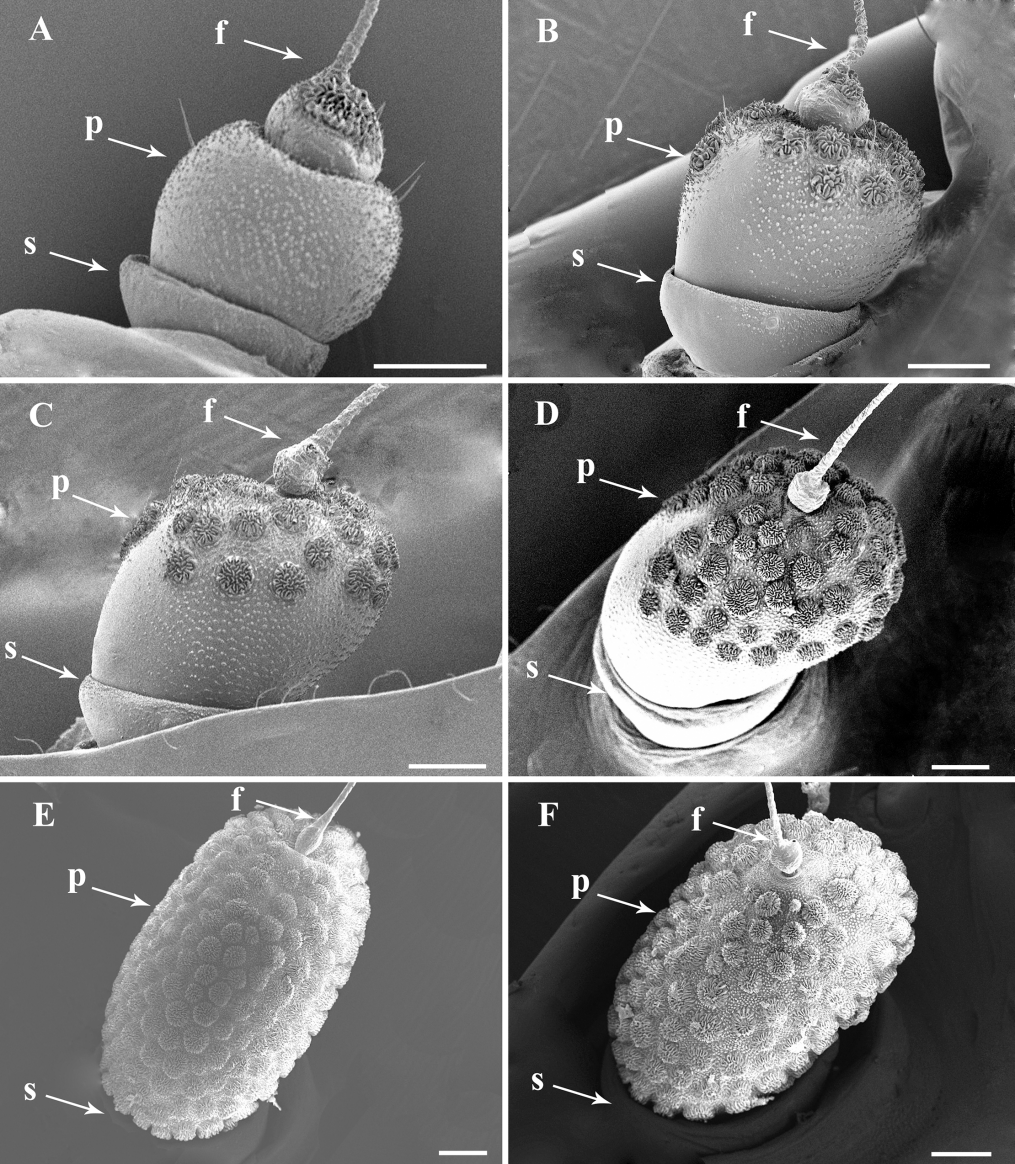
General view of the three-segmented antenna of *Lycorma delicatula* nymphal instars and adults. (A–D) Full antenna first to fourth instar, respectively. (E–F) Full antenna in adult female and male, respectively. Scale bars: A = 90 μm; B–F = 100 μm. Abbreviations: f, antennal flagellum; p, antennal pedicel; s, antennal scape.

**Fig 2:**
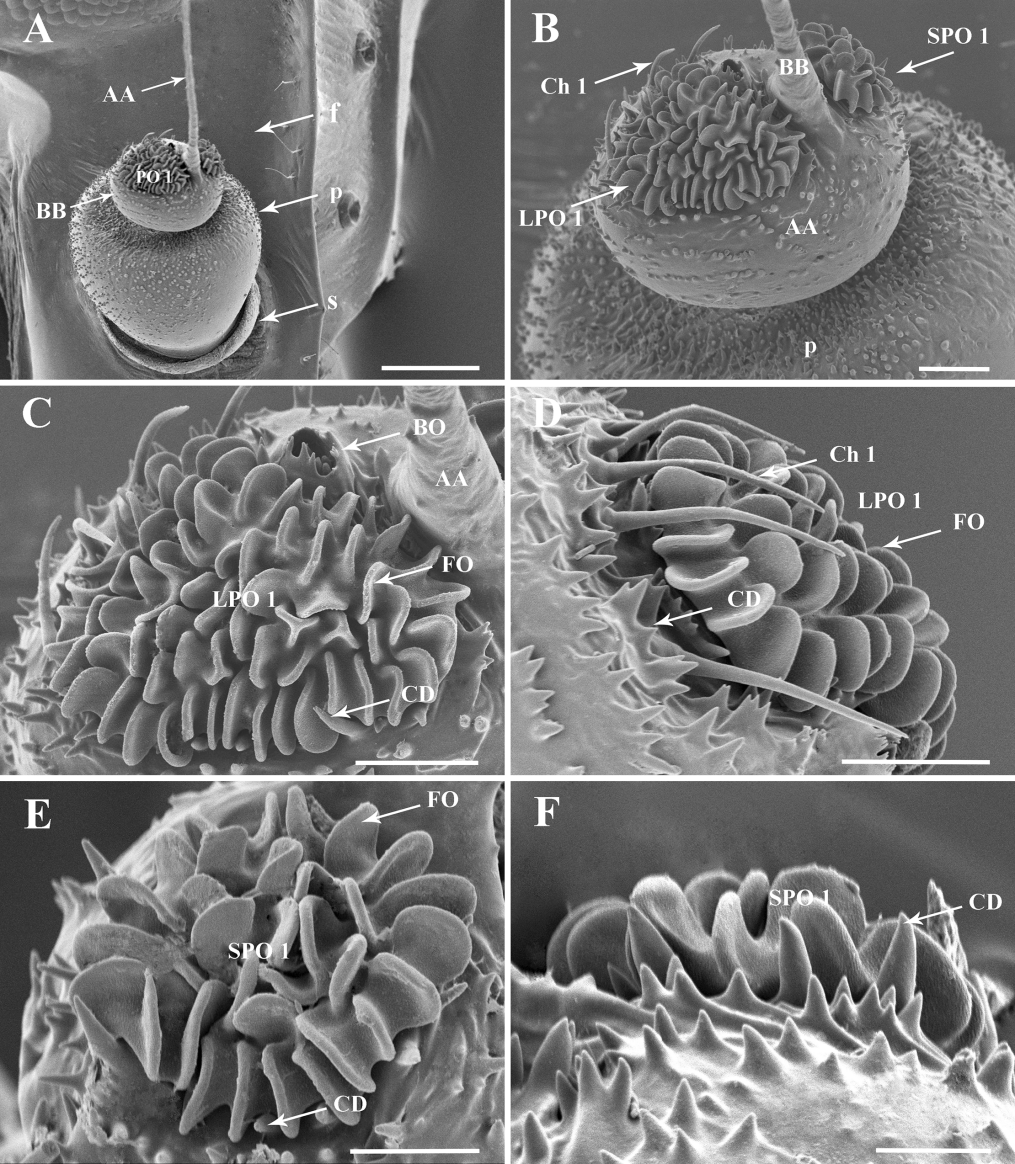
Details of the antenna of the first nymphal instar of *Lycorma delicatula*. (A) General view of the antenna, showing the distribution of sensilla on antennal surface. (B) Basal bulb of antennal flagellum with LPO 1 and SPO 1. (C–D) LPO1 and opening of Bourgoin's organ. (E–F) SPO 1, showing the multiporous folds and cuticular denticles with smooth surface. Scale bars: A = 100 μm; B = 25 μm; C = 20 μm; D = 20 μm; E = 15 μm; F = 10 μm. Abbreviations: AA, apical arista of antennal flagellum; BB, basal bulb of antennal flagellum; BO, Bourgoin’s organ; CD, cuticular denticles; Ch 1, sensillum chaeticum type I; f, flagellum; LPO1/SPO1, large/small plate organ type I; p, pedicel; s, scape.

**Fig 3:**
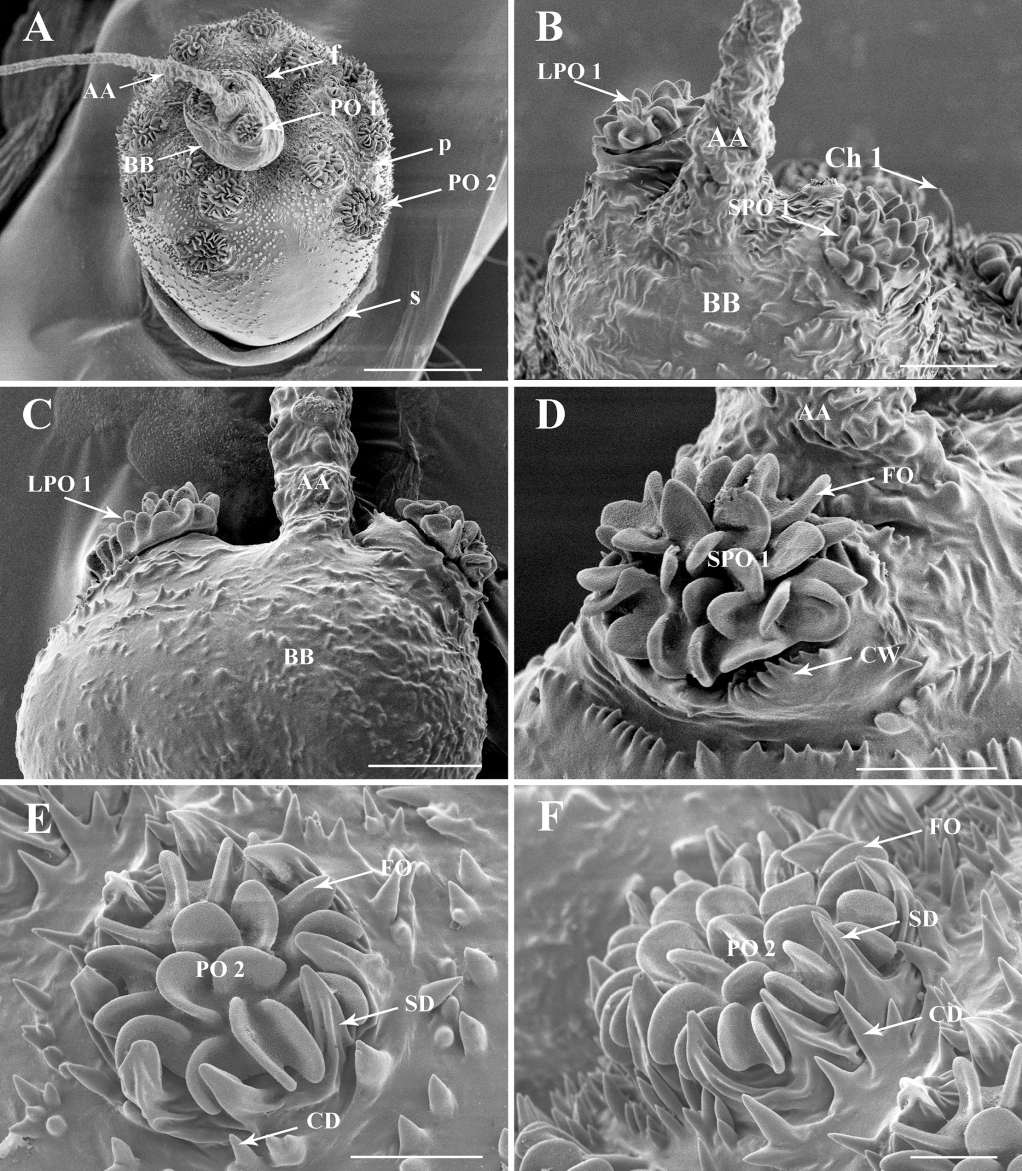
Details of the antenna of the second nymphal instar of *Lycorma delicatula*. (A) General view of the antenna, showing the distribution of sensilla on antennal surface. (B–C) Basal bulb of antennal flagellum, LPO1 and SPO1. (D) SPO1 with an indented and raised wall. (E–F) Plaque organ type II on the pedicel, showing the multiporous folds and ridged sclerotized denticles. Scale bars: A = 100 μm; B and C = 25 μm; D and E = 15 μm; F = 10 μm. Abbreviations: AA, apical arista of antennal flagellum; BB, basal bulb of antennal flagellum; CD, cuticular denticles; Ch 1, sensillum chaeticum type I; CW, encircling walls; f, flagellum; FO, folds; p, pedicel; PO 1, plate organs type I; PO 2, plate organs type II; s, scape; SD, sclerotized denticles.

**Fig 4:**
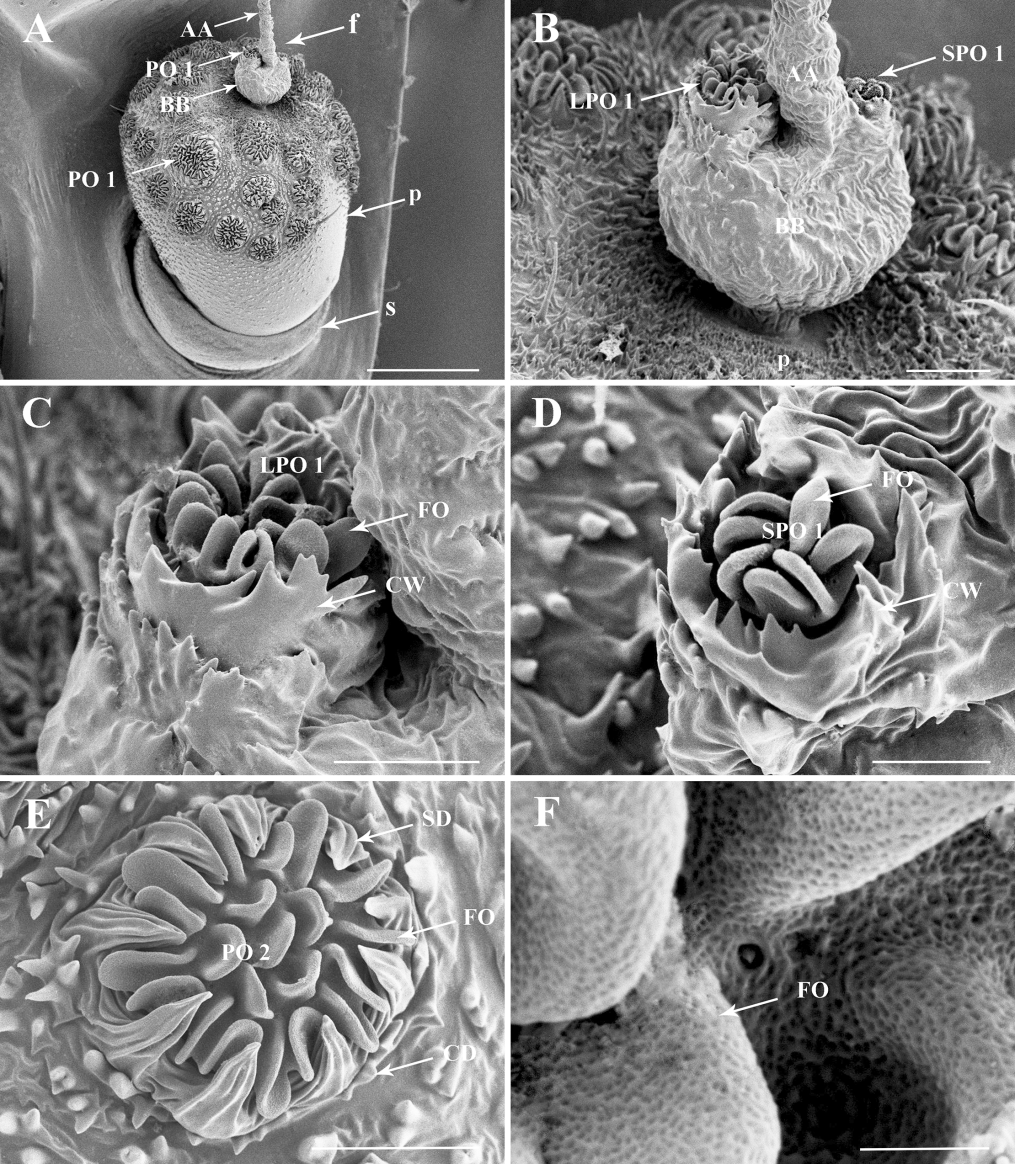
Details of the antenna of the third nymphal instar of *Lycorma delicatula*. (A) General view of the antennae, showing the distribution of sensilla on antennal surface. (B) Basal bulb of antennal flagellum, LPO 1 and SPO1. (C) LPO 1 on the anterior faces of the antenna. (D) SPO1 on the posterior face of the antenna. (E) PO 2 of the pedicel. (F) Multiporous surface of PO2 lobes. Scale bars: A = 150 μm; B = 25 μm; C = 15 μm; D = 10 μm; E = 20 μm; F = 2 μm. Abbreviations: AA, apical arista of antennal flagellum; BB, basal bulb of antennal flagellum; CD, cuticular denticles; CW, encircling walls; f, flagellum; FO, multiporous folds; p, pedicel; PO1, plaque organ type I; PO2, plaque organ type II; s, scape; SD, sclerotized denticles.

**Fig 5:**
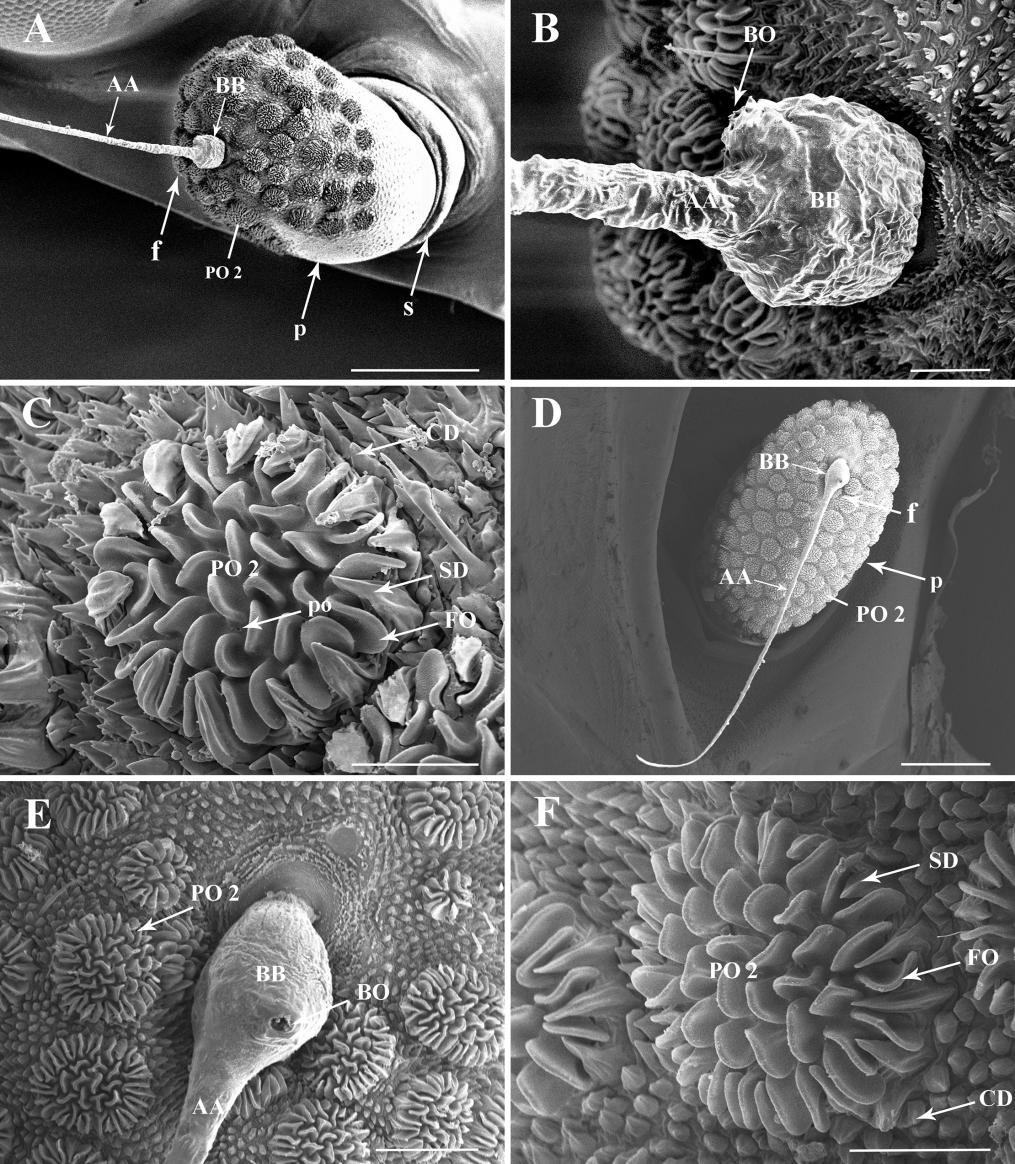
Details of the antenna of fourth nymphal instar *Lycorma delicatula* and in adults. (A) General view of fourth nymphal instar antenna, showing the distribution of sensilla on antennal surface. (B) Basal bulb of antennal flagellum in fourth instar, PO 1 are absent. (C) PO 2 on the pedicel of the fourth instar, some pores (po) are visible. (D) General view of adult antenna, showing the distribution of sensilla on antennal surface. (E) Basal bulb of adult antennal flagellum. (F) PO 2 in adult pedicel. Scale bars: A = 250 μm; B = 25 μm; C = 20 μm; D = 250 μm; E = 50 μm; F = 25 μm. Abbreviations: AA, apical arista of antennal flagellum; BB, basal bulb of antennal flagellum; BO, Bourgoin’s organ; CD, cuticular denticles; f, flagellum; FO, folds; p, pedicel; PO 2, plaque organ type II; s, scape; SD, sclerotized denticles.

Antennae of *L*. *delicatula* are located laterally on the head capsule. They consist of three distinct segments: a short ring-like scape (s), a prominent pedicel (p) and a pseudo-segmented flagellum (f). The scape connects the antenna to head capsule and has few sensilla (Fig 1), while the pedicel is covered with sensory units in first to fourth instars and adults. The flagellum is composed of two distinct portions, an expanded basal bulb (BB) and a threadlike apical arista (AA). The basal bulb is inserted basally in a socket on the apex of the pedicel. Apically it presents a subapical opening encircled by concentrically arranged cuticular denticules (CD) (Figs 2A, 2B, 3A, 3C, 4B and 5B) indicating the location of the internal Bourgoin sensory organ (BO), which is flanked by the long arista, sharply ending apically (Figs 1, 2A, 2B, 3A, 3C, 5E, 4A, 4B, 5A, 5B and 5D).

**Fig 6:**
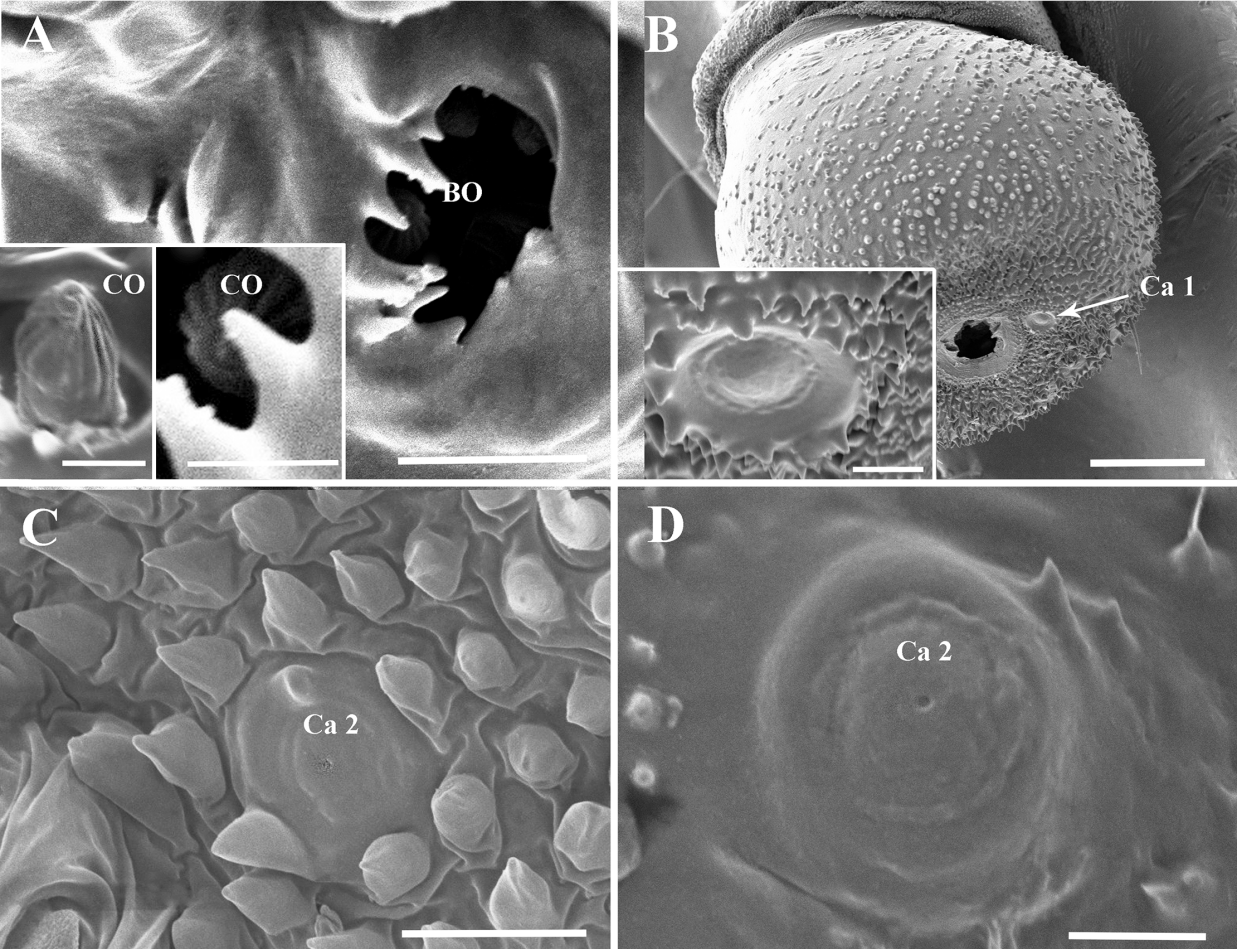
Bourgoin’s organ and campaniform sensilla on the antennae of *Lycorma delicatula*. (A) Bourgoin’s organ aperture on basal bulb of flagellum and in boxes sensillum coeloconicum inside. (B) Ca 1 on apex of antennal pedicel. (C) Ca 2 on antennal pedicel. (D) Ca 2 on antennal scape. Scale bars: A = 5 μm (2 μm in box); B = 50 μm (5 μm in box); C = 10 μm; F = 5 μm. Abbreviations: BO, Bourgoin’s organ; Ca 1, campaniform sensillum type I; Ca 2, campaniform sensillum type II with a median pore.

**Fig 7:**
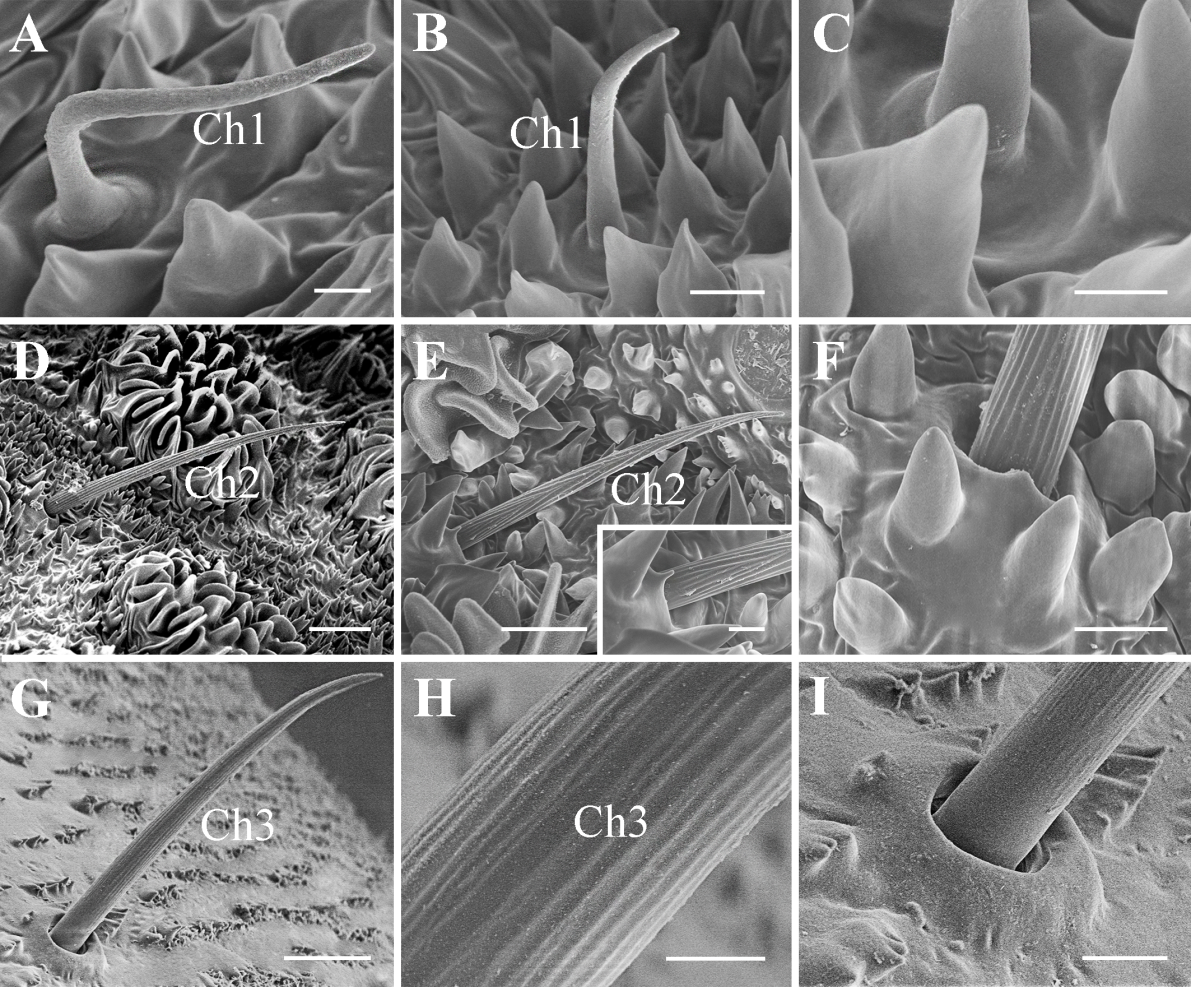
Three types of chaetica sensilla on the antennae of *Lycorma delicatula*. (A)–(B) Ch1 on the pedicel, with length varying from 20 to 35.5 μm. (C) Magnification of Ch1 base. (D) CH2L and (E) CH2 on the pedicel, the box in (E) showing the magnification of socket base and convergent ridges. (F) Magnification of Ch2L socket base and mostly parallel ridges. (G) Ch3 on antennal scape. (H) Magnification of longitudinally ridged shaft. (I) Magnification of Ch3 socket base. Scale bars: A, C, H = 2.5 μm; B, F, I = 5 μm; D = 15 μm; E = 10 μm (2.5 μm in the box); G = 20 μm. Abbreviations: Ch 1, 2, 3, sensilla chaetica type 1, 2, 3.

Four types of antennal sensory units can be observed. These are sensilla chaetica (Ch), campaniformia (Ca), coeloconica (Co), and the plate organs (PO), divided into subgroups according to their morphological characteristics (Table 1). Two subtypes of plate organs, referred as PO1 and PO2, (LPO1, SPO1 respectively for the large and the small plate organ of the flagellum), were separated on the basis of the conformation of their external cuticular denticule row. They are distributed on the flagellar basal bulb and on the pedicel, respectively. Coeloconic sensilla (Fig 6A) are only present inside the Bourgoin’s organ on the flagellar basal bulb. Two subtypes of dome shaped sensilla or campaniform sensilla were named Ca1 and Ca2 (Fig 6D–6F). Four subtypes of sensilla chaetica (Ch1, Ch2, Ch2L, Ch3) were recognized (Fig 7), also based on the ratio of their length to their basal diameter (Table 1). Characteristics, locations, and measurements of these sensilla are given in Tables 1, S1 and S2 Appendices.

### Sensilla chaetica Type I (Ch1)

Sensilla chaetica Type I are smooth sensilla with no pore visible (Figs 2B, 2D, 3B, 7A and 7C). Their base is distinctly elevated inside a rounded cupula and separated from the shaft by a tiny circular sulcus. They are probably basally flexible. They are basally straight and faintly curved at mid length, and blunt-tipped. They are robust sensilla of 20–35 μm long, with a basal diameter of 3.5–6.6 μm, and a characteristic L/BD ratio = 4.5–6.6 (Table 1).

They surround the flagellar plate organ LPO1 in the first instar and thus are present on the dorsal side of the flagellar base. In the second instar, one sensillum is found on the flagellar base and several are scattered on the apex of the pedicel. In all other instars these are mainly distributed in the distal part of the pedicel but occasionally occur on the medial and proximal part. They are probably true mechanoreceptive sensilla trichodea.

### Sensilla chaetica Type II (Ch2 and Ch2L)

Sensilla chaetica Type II (Ch2) are long and slender sensilla inserted into a raised socket base with a basal pore, with a surface ridged with straight longitudinal grooves, and acute tipped (Fig 7D–7F). They are most often regular faintly curved sensilla of 38–56 μm long, 2.45–3.4 μm basal diameter, and with a characteristic L/BD ratio = 15–19 (Table 1). They are scattered on the antennal pedicel of all nymphal stages and also present in adult females (S1 Appendix). A longer type (Ch2L) is present on pedicel on 4th instar and adults: 75–78 μm long, with a 4.2 μm basal diameter and with a characteristic L/BD ratio = 17.8–18.6 (Table 1). They are probably chemo-mechanoreceptive chaetica sensilla.

### Sensilla chaetica Type III (Ch3)

*Sensilla chaetica* Type III (Fig 7G–7I) are medium size chemo-mechanoreceptive sensilla similar to Ch2 but more robust, vertically inserted into a raised socket, and slightly bent only apically (Table 1) and acute-tipped. Its shaft is longitudinally ridged with more ridges than Ch2. This sensilla is 20–32 μm long and 2.2–3.3 μm wide at the base. It is characterized by a L/BD ratio of 9.5–9.6 (S1 Appendix). A single Ch3 is present in all stages on the latero-ventral side of the scape. They are probably chemo-mechanoreceptive chaetica sensilla.

### Plate organs Type I (PO1)

*L*. *delicatula* type I plate organs (Table 1) are of the crenelated or lobulated ridged plate type [24, 25]. The plate surface is developed into lobe-like apically rounded expansions, most generally single but a few are Y-shaped (Figs 2, 3A, 3D, 4A and 4D). External cuticular denticles at the periphery are weakly developed, not surpassing plate lobes, basally conical, and 1.5 times longer than the external cuticular denticles found elsewhere on the antenna.

A pair of these PO1 (Fig 2) occurs in first to third instars only on the flagellum basal bulb. In first instars, the larger PO1 (LPO1) is situated anteriorly beside Bourgoin’s organ, circled with 17–21 cuticular denticles and bearing about 68–69 lobe-like expansions. The smaller PO1 (SPO1), is situated posteriorly with some 27–28 lobe-like expansions surrounded by 9–11 external cuticular denticles. In the second instar, PO1 cuticular denticles are absent. The base of the plate organ is distinctly elevated over the pedicel surface and a circular furrow encircling the sensory unit is clearly visible. The surrounding external cuticular denticles are organized into elevated comb-like ridges. In the third instar these ridges are more developed and connected together to fully surround the plate organ (Fig 4C and 4D). PO1 are absent in fourth instars and in adult of *L*. *delictula* (Fig 5). From first to third instar, the PO1 strongly decrease in size (70, 34, 22 μm and 40, 24, 12 μm for LPO1 and SPO1, respectively) and in number of lobe-like expansions (LPO1: 68-27-14 and SPO1: 27-16-7).

### Plate organs Type II (PO2)

Plate organs subtype 2 (Figs 4E, 4F and 5; Table 1) are also of the lobulated ridged plate type with the plate surface developed into 22–45 lobe-like expansions that are apically rounded. Lobes are organized in a first peripheral concentric row of single lobes (from 10 to 30 in the larger units) with a central area where lobes are less organized and more often Y-shaped. Peripheral external cuticular denticles (7–24 according to plate organ size) are strongly developed, widely sulcated, with a robust and wide triangular base and apically pointing and directed concentrically.

**Fig 8:**
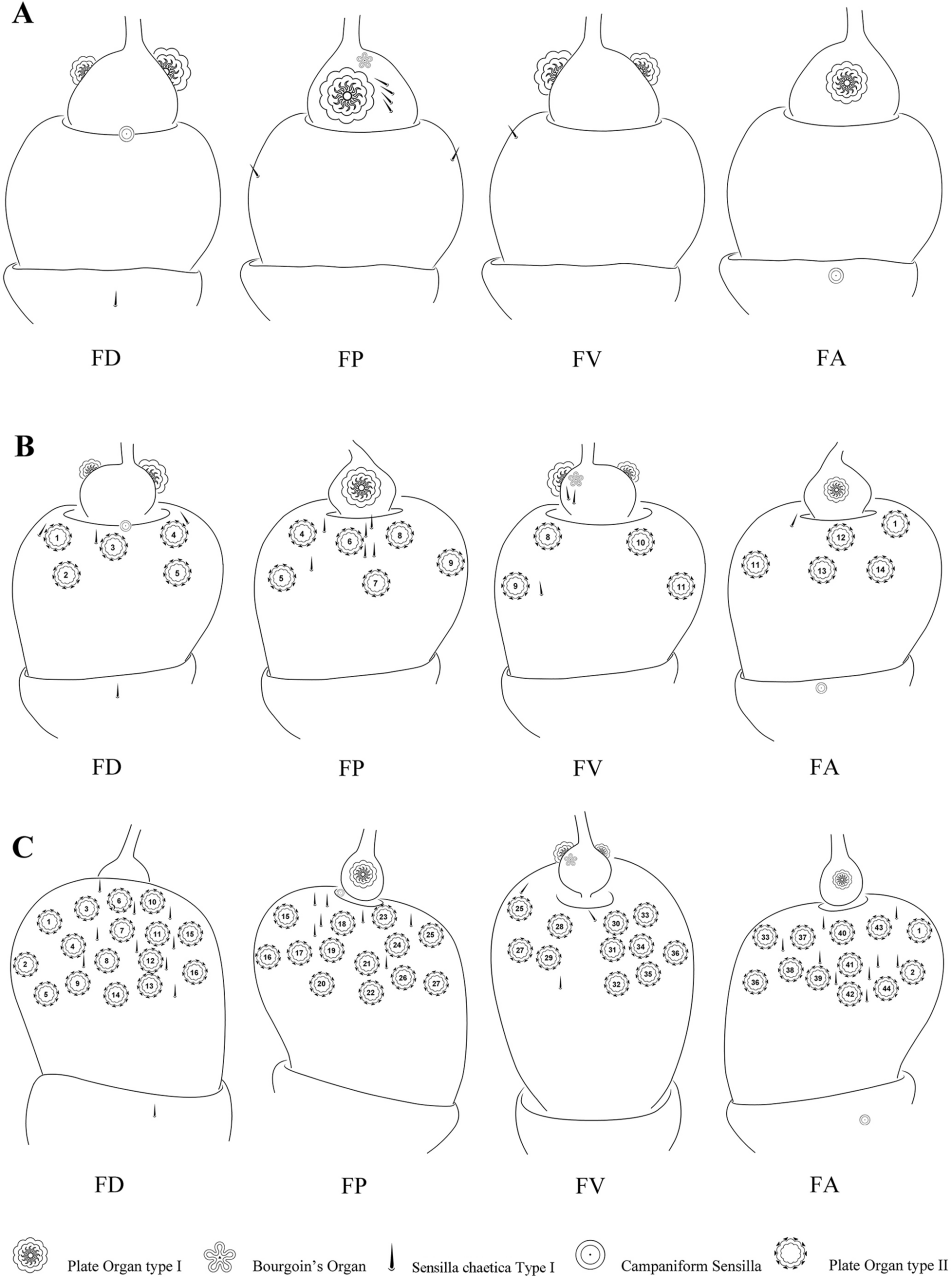
Distribution of sensilla on first to third instar nymphal antennae of *Lycorma delicatula*. (A–C) First–third instar nymphal antennae, respectively. Abbreviations: FA anterior face; FD dorsal face; FP posterior face; FV ventral face.

**Fig 9:**
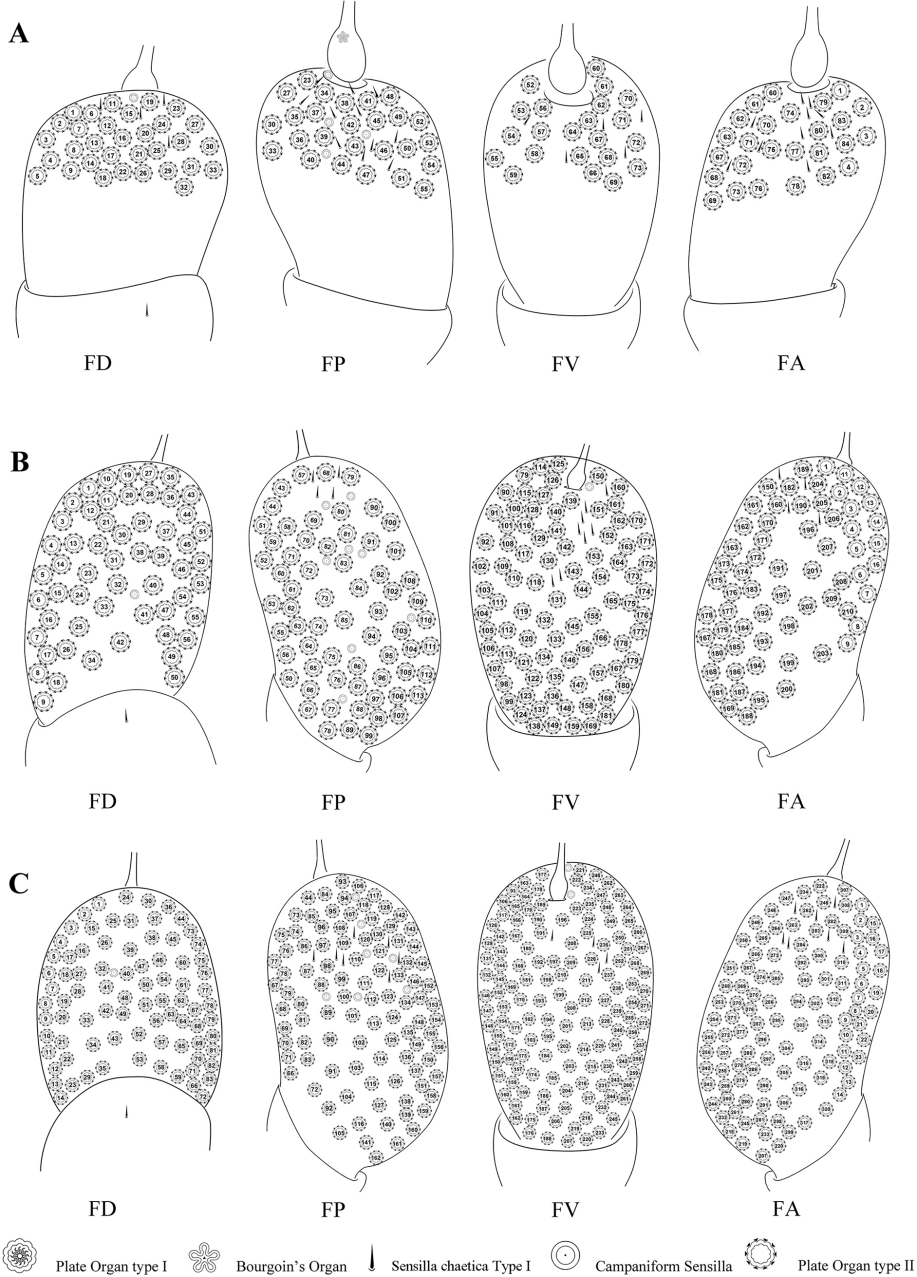
Distribution of sensilla on forth instar nymphal and adult antennae of *Lycorma delicatula*. (A) Forth instar nymphal antennae (B) Male antenna. (C) Female antenna. Abbreviations: FA anterior face; FD dorsal face; FP posterior face; FV ventral face.

PO2 are absent in first instar but present in all other stages on the pedicel in a remarkable variety of size, number and distribution, which increases from second instars to adults (Figs 2–5, 8 and 9). In the second instar, they are separately distributed on the distal region of the pedicel with gradual higher density towards the apical region. In the third and fourth instar, PO2 are uniformly scattered on the distal and middle regions, except for a triangular bare area on lateral side; they are densely distributed all over the pedicel in adults.

With each stage starting in the second instar (S1 and S2 Appendices), PO2 generally gradually increase in number (n = 14, 44, 84, and 210 in adult males, 320 in adult females), in size (Ø = 39.55, 44.79, 51.83, 52.71 μm in adult females—in adult males they are however smaller (Ø = 44.96 μm) than in fourth instar), and in complexity with the number of lobes per plate in nymphal instars (n = 22, 28, 37). In adults however, the number of lobes appears to be less important (n = 31, 36 respectively in males and females). No noticeable increase of lobe size was noted from third instars to adults (10 μm). From second to fourth instar, the dorsal surface of the antenna carries the most important number of PO2 (DF = 5, 16, 32) and the ventral side the least (VF = 4, 11, 22) (S2 Appendix). This distribution is totally reversed in adults (DF = 56, 83 and VF = 88, 138) in male and female respectively. A strong sexual dimorphism is observed in the number and the size of PO2 in adults (S2 Appendix).

### Evolution of the plate organs sensory surface

**Fig 10:**
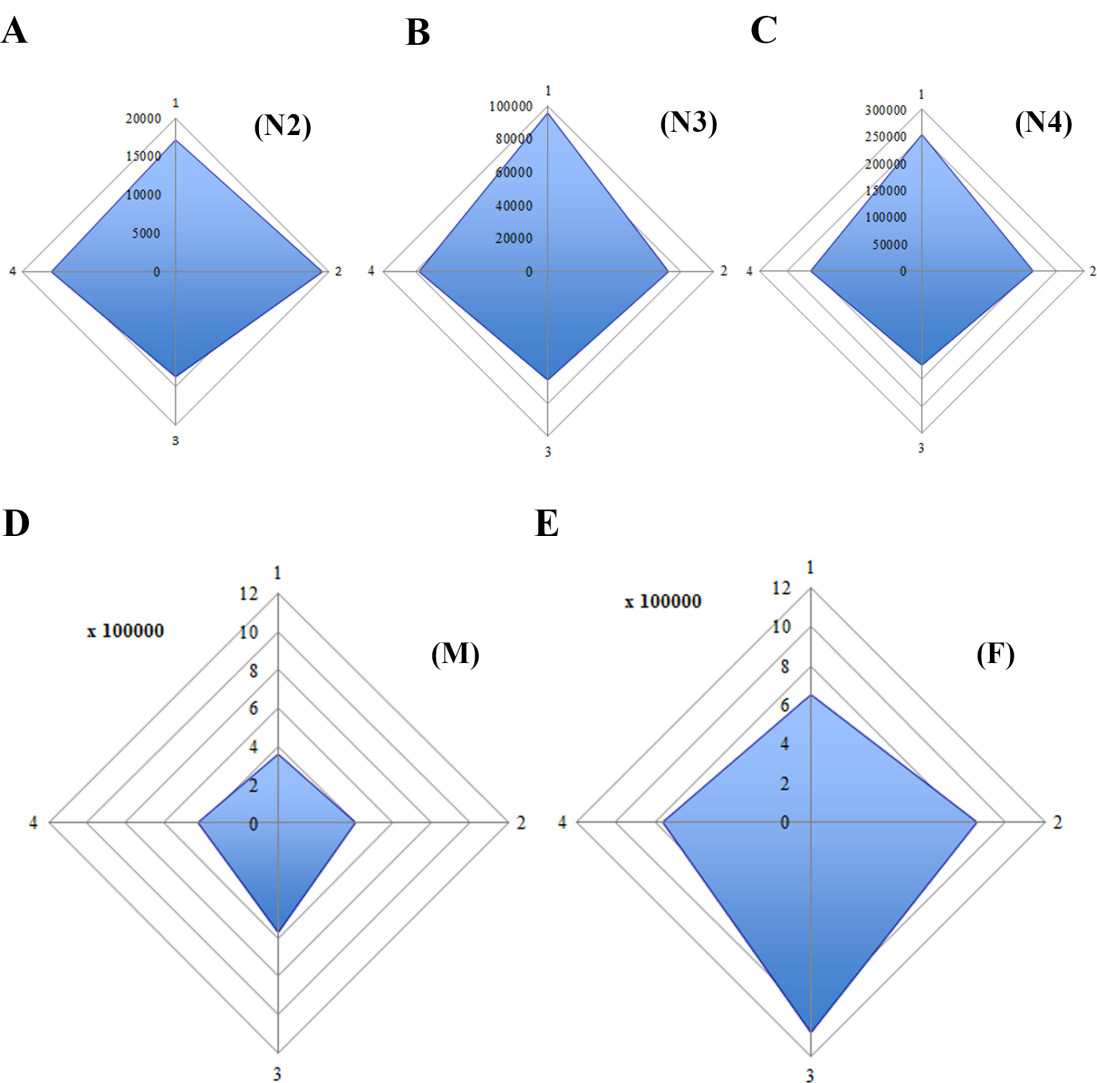
Proportional representation of sensory surface of *Lycorma delicatula* antennae in nymphal instars and adults (for one antenna, in μm^2^). Abbreviations: 1, dorsal face; 2, anterior face; 3, ventral face; 4, posterior face of the antenna; F, female; M, male; N2 –N4, second to fourth instar.

With the distribution of plate organs at the different stage (Figs 8 and 9), the sensory surface (SS) of the sensory plate organs in the different nymphal instars and in male and female adults was estimated for the total surface of one antenna and per antenna face (S2 Appendix). Following the number of plate organs, the sensory surface increases on each pedicel face with the nymphal instar and is more important on the dorsal face (Fig 10). In adults it is greater on the ventral face and a strong sexual dimorphism is observed (SS = 1.36 mm^2^ and 2.51 mm^2^ in male and female respectively) (Fig 10). Between instars from first to fourth, the sensory surface increased respectively by a factor of 2.6, 5.2 and 2.5 and between fourth instar and adult stage by 2.0 and 3.8 for males and females respectively (S2 Appendix). The major increases occur between second and third instar, and between fourth instars and adult females. From the first instar, the sensory surface area increases 67.7 times in males and 125.2 times for females.

### Sensilla campaniformia (Ca)

Sensilla campaniformia (Fig 6D–6F) are proprioceptor aporous dome-shaped sensilla. Two subunits exist based on the presence or absence of a central cupule and sensilla diameter. Type 1 (Ca1) is the largest at 12.5–18.3 μm diameter. It size vary little with instar but clearly smaller in male than in female in the adult stage (S1 Appendix). One sensillum occurs near the apical portion of the pedicel near the basal flagellar bulb and another sensillum on the ventral surface of the scape. In fourth instars and adults, other sensilla campaniformia (Ca2) occur on the dorsal and posterior surface of the antennal pedicel (Fig 6D and 6E) and scape (Fig 6F). A distinctive cupule occurs at the centre of the dome. They are slightly smaller than Ca1 and have a diameter of 9.99–11.50 μm.

### Coeloconica sensilla (Co) and Bourgoin’s organ (BO)

An evident BO with an aperture surrounded by petal-like walls occurs on the expanded basal bulb (Figs 2C and 6A) at each nymphal stage and in adults. Sheltered by the walls and only observable looking straight down in BO, three cone-shaped coeloconica sensilla sculptured with straight longitudinal grooves are present (Fig 6A).

## Discussion

Antenna sensilla of *L*. *delicatula* are present in all life stages. They have a remarkable diversity of shapes, sizes, numbers, and distribution. They occur and evolve differently with nymphal instars, and their distribution also differs between adult males and females (Table 1 and S1 Appendix). Four main types of sensory units can be observed: hair-like sensilla (chaetica and trichodea), campaniformia, coeloconica, and plate organs. They can be divided into subgroups according to their morphological characteristics. For sensilla chaetica / trichodea, the ratio of the length to basal diameter appeared a better way to clearly separate subgroups as the length criterion only was often not enough for clear discrimination. From a morphological point of view, several new findings need to be addressed before trying to correlate these results with some behavioural aspects of *L*. *delicatula*.

### Sensory units of the planthopper flagellum

As in other planthoppers, the flagellum has the distinctive Bourgoin's organ [30], a complex sensory structure first described in Tettigometridae, also reported as the 'basal sensory organ' [31, 32], or 'flagellar atrium' [33]. Initial histological observations [31] revealed that three chambers roughly in series formed Bourgoin's, which opens exteriorly by a single aperture while the two deeper chambers contain a different type of sensilla probably coeloconic. In a TEM study of the cixiid *Hyalesthes obsoletus* Signoret [34] 2 chambers were observed, also with a single external aperture. In the first chamber a double-walled coeloconic sensillum is present and in the second chamber there is a grooved-peg coeloconic sensillum. The first sensillum is a possible thermo- and olfactory receptor and the second is a CO_2_ receptor [34]. The present study suggests that Bourgoin's organ is probably present in the first instars of all planthoppers and not only restricted to adult planthoppers and that it is probably slightly variable in his general conformation.

The occurrence of chemoreceptive sensory plate organs on the expanded basal bulb of the nymphal flagellum is of particular interest and reported for the first time in planthoppers although they have been described previously but misinterpreted as either a nymphal conformation of the Bourgoin's organ [31] or even as a reduced flagellum arista [32]. They are morphologically distinct from those on the pedicel.

In some adult planthoppers there is an additional complex sensory structure on the flagellum referred to as the 'antennal second projection' [35, 36] (also been reported as 'arista' [37], 'basal flagellar process' [38] or 'cuticular spur' [34]). In the cixiid *H*. *obsoletus*, this it is a process supporting three sensilla styloconica that could function as thermo-hygroreceptors [34] but various shapes of this structure have been observed in species from several planthopper families [38]. It is, however, not general in planthoppers and it is clearly absent in *L*. *delicatula*. The basal flagellar process represents an independent, new, and complex sensory structure as the Bourgoin's organ, and it requires new observations in nymphs of species where it has been mentioned. Particularly it should be investigated more precisely for its conformation and how it could not be related (homologous) to one of the nymphal flagellar plate organs. A better knowledge of the distribution of this structure within all planthopper families would also help explain if it is, as Bourgoin's organ, a basic and distinctive structure of the Fulgoromorpha ground plan. Species lacking the structure might then represent cases of independent secondary loss or reduction.

With the presence of numerous and diverse sensilla, the antenna flagellum is considered to carry the main sensory function in most insects [17]. In planthoppers however, it has been reduced to a thin arista—probably secondarily segmented [24], being carried by a basal flagellum swelling notably bearing a few sensilla in adults [31, 34]. It is therefore probable that sensory units originally developed on the Hemiptera flagellum were lost with planthopper flagellum specialization. The presence of the 2 nymphal flagellar POs, still carried by the basal flagellar swelling in first three instars, should reflect this trend of an evolutionary simplification of the antennal flagellum but not fully affecting the first flagellomere in first instars. This supports the heterochrony hypothesis [39] that 'imaginilization' (= progenesis) of the Cicadina (= Auchenorrhyncha) flagellum started from its apex by early nymphal thinning and polymerisation of the flagellum. This process may have occurred, to various degrees, in the different Auchenorrhyncha lineages. The function of plant exploration (close chemo- and tactile recognition) is usually accomplished by long antennae in phytophagous insects. In planthoppers, due to reduction of the flagellum, recognition was transferred to the apex of the labium during dabbing of the plant surface [27, 28].

### Sensory plate organs of the planthopper pedicel

However, an important antennal chemosensory function in planthoppers remains concentrated on the antennal pedicel with the unique development of numerous complex sensory plate organs that, with other sensilla, may provide longer distance host-plant recognition [21–23].

Planthopper sensilla disparity was first recognized by Bugnion [40] and only few early studies addressed this topic [21, 22, 40–43]. Since, several taxon-specific studies recently reviewed [44] have been published, revealing taxonomic specificity at various level of the planthopper classification The high diversity of these structure now described allows to adopt now a new classification from the previous ones [25, 26] with only 3 major types of sensilla placodea according to their general morphology: 1) a *free-tip type* with a multi-digitized or setae-like projected plate than can lose its erect setae-like conformation for a flat conformation or a flattened star-shaped plate, 2) a *merged-tip type* with a circular central plate of irregular ridged surface previously described as Y-shaped, thorn-like, lobulated or crenelated (notched) type to which *L*. *delicatula* belongs as all Fulgoridae [44, 45], and 3) a *no-tip type* with a central plate always flattened, not ridged nor lobulated, with a circular or clover-leaf like circumference.

In planthoppers, sensilla placodea are present in numbers ranging from 12 in the tropiduchid species *Ossoides lineatus* Bierman [41] to hundreds in *L*. *delicatula* (with an average of 210 and 320 sensilla placodea, respectively, in males and females). Other Fulgoridae [41] or Tettigometridae [31] have also large numbers of sensilla placodea. More than 1000 sensilla were found in the fulgorid *Fulgora castresii* Guérin-Méneville and in some Derbidae species (Bourgoin, unpublished data). In planthoppers, these complex sensory structures are limited by a circular furrow and characterized by a central multi-porous plate of various shapes and, when present, by internal strong cuticular denticules [31]. TEM studies have shown that the sensillum center has a different cuticular organisation than the cuticular denticules: thinner (150 μm) and multiporous, versus 500 μm and aporous respectively) [23]. External to the circular furrow, differently shaped cuticular denticles might also arrange circularly around the sensillum [31].

During the different development stages of *L*. *delicatula* the number of pedicel plate organs gradually increased (ranging from 0, 14, 44, 84 and, respectively, 210 and 320 in males and females) (S2 Appendix). Between the second and the fourth instar the number of sensilla placodea only increased by a factor of 6 but this number increased but by 15 and 23 in males and females respectively, suggesting a more important or possible specific function of these sensory structures in the adults than in the nymphs. Particularly in the third and fourth instar, sensilla placodea are one third more numerous on the pedicel dorsal face (16 and 32 respectively) than on the ventral face (11 and 22 respectively). This observation is totally reversed in adults (dorsal face (FD) = 56, 83 and ventral face (FV) = 88, 138 respectively in males and females) with the pedicel FV having 25% more sensilla than the FD. While specific locations and symmetrical arrangement of these sensilla has been recorded for some delphacid taxa [24] this is not the case in *L*. *delicatula*.

**Fig 11:**
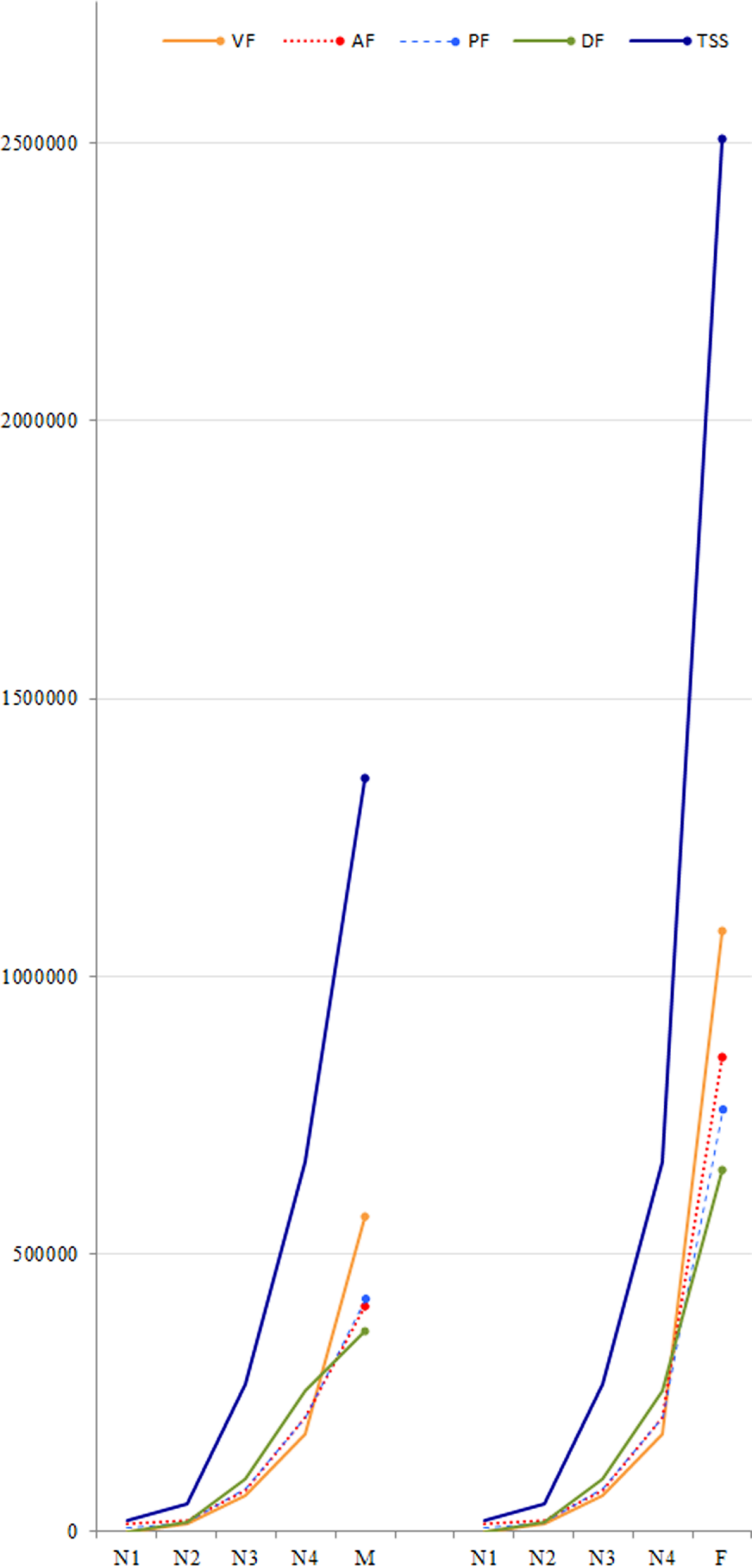
Development of sensory surface of plate organs on antennae in nymphal instars and adults (for one antenna, in μm^2^). Abbreviations: FA, anterior face; FD, dorsal face; FP, posterior face; FV, ventral face; TSS, total sensory surface.

The corresponding sensory surface (SS) of the sensory plate organs during the nymph and adult stages was estimated for the total surface of 1 antenna and per antenna faces (S2 Appendix). Because not only the sensilla placodea increase in number but also in size and in the number of sensory lobes, their functional importance is best estimated by the amount of sensory surface that these sensilla provide to the insect. Accordingly, this sensory surface increases on each pedicel face with each nymphal instar and is more important on the dorsal face. In adults, it is more important on the ventral face and a strong sexual dimorphism is observed (Figs 10 and 11): indeed the complete sensory surface area provided by the sensilla placodea by the antenna pair exceeds 2.7 mm^2^ in males and 5 mm^2^ in females. In contrast, the body length of male adults is only 17–20 mm and 20–25 mm in females [4]!

### Relating antennal sensilla diversity and possible specific functions

**Fig 12:**
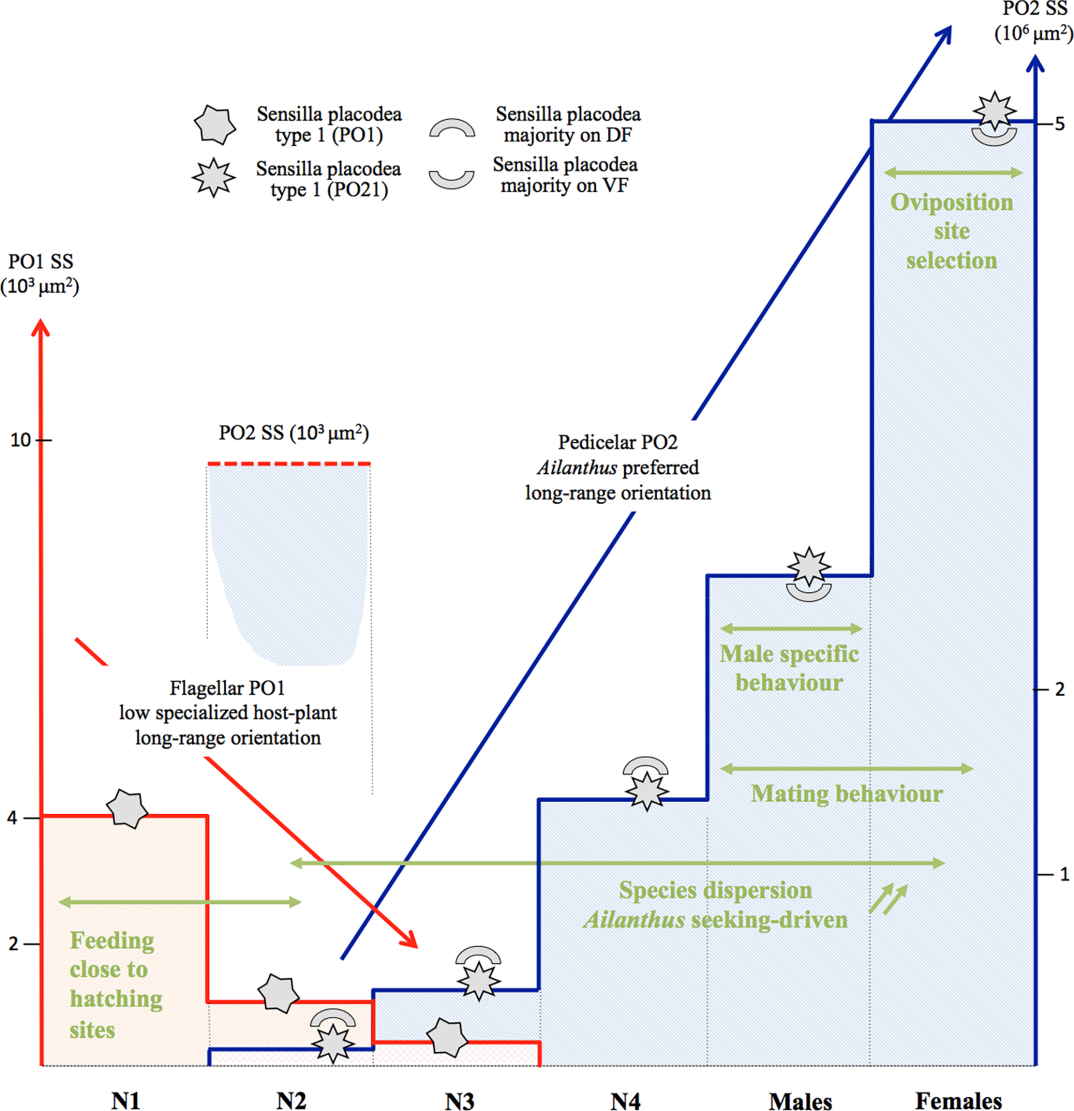
Diagram of antennal plate organs and behaviours, showing their evolution and possible functions in one cycle of *Lycorma delicatula*. Sensory surfaces for flagellar plate organs type I (PO1) in red (scale 10^3^ μm^2^), and for pedicel plate organs type II 2 (PO2) in blue (scale 10^6^ μm^2^). PO2 in N2 added for comparison with PO1 at same scale (doted line). Abbreviations: DF, VF: dorsal, ventral face of antenna; SS sensory surface.

All the recognized types and groups of sensilla are clearly correlated with their distribution on the antenna of the various instars (Table 1): flagellar plate organs of type 1 are absent in fourth instars and adults, and pedicel plate organs of type 2 are absent in first instars; sensilla chaetica type Ch2L seem only present in 4th instar and adults; sensilla chaetica of type 3 are generally distributed in all instars and sensilla coelonica are concentrated within the Bourgoin's organ. These observations depict a selective distribution of the sensilla in time and space and suggest specialized functions of the different parts of the antenna consistent with the age of the insect. Porous and multiporous sensilla chaetica, coelonica, and placodea have been shown having a chemoreceptive functions [21–23, 29, 34] and may participate in host-plant recognition. Subsequently, these olfactory sensory structures might be involved in selective behavioural responses. In this respect, and assuming that the sensory functions of these sensilla remain identical during the different life stages, the results suggest various hypotheses:

1. The progressive increase of the pedicel plate organ number during nymphal stages and the greater than 100% increase of the functional sensory surface in adults, suggest that the olfactory function is progressively installed in nymph and optimally reached in the adult stage only (Fig 12). It supports previous observations such as essential oil lavender stimuli only start to be repellent effective in the third instar in *L*. *delicatula* [46] or oil spearmint stimuli as attractant effective in fourth instar [47]. As morphological structures are progressively built in heterometabolic insect for adult function (reproduction, flight …), a progressive development of antennal sensory units in this species allows gradual development for more specific imaginal functions.

2. The unexpected shift of the pedicel sensory surface from the dorsal to the ventral location in adults supports also the previous hypothesis and suggests significant changes in associated behaviours (Fig 12). The sensory surface is one third more important in pedicel dorsal face than in the ventral one in nymphal stages (Fig 10, S2 Appendix) facing most of what occurs directly around the nymph. This would assist them in a better host-plant orientation in their biotope. In reverse, in adults the sensory surface is almost twice as important in the pedicel ventral face than in the dorsal one: 37% and 40% in males and females respectively (Figs 10 and 11, S2 Appendix) and most of plate organs directly face the host-plant. This might assist adults in a better and closer orientation towards substrate sources of olfactory stimuli. As symmetrical arrangements of few antennal sensilla in some delphacids were hypothesized to perform olfaction [24], it is a greater number of sensilla on the ventral side of the antenna that might have been selected for this task in *L*. *delicatula*.

3. Chemoreception involving the plate organs is absent from the pedicel in the first instar but is supported by two unique and morphologically specific plate organs of the flagellum (PO1) (Fig 12). This first instar flagellar function seems to remain however minimal as already in the second instar, even with few pedicel plate organs (PO2), the pedicel already represents 94% of the total functional sensory surface (S2 Appendix). Accordingly, during the second instar, the plate organ olfactory function shifts from the flagellum to the pedicel. As first instar of *L*. *delicatula* have been notably observed to be polyphagous [3, 48], we suggest that this shift might occur with functional modifications (supported by the morphology of two different types of sensilla) that may be involved with a more selective diet spectrum and preference for *Ailanthus* as it has been observed in the following instars [3].

4. A significant sexual dimorphism occurs in the numbers of sensilla placodea in male and female *L*. *delicatula* (Fig 12). Such a case was previously unknown in planthoppers [24, 49], while differences in responses to plant volatiles between sexes were already observed in the rice brown planthopper *Nilaparvata lugens* (Delphacidae) [22] even associated with sex specific behaviours, in the cixiid *H*. *obsoletus* [23]. Obviously the sensilla placodea olfactory function play a more important role for females than for males in *L*. *delicatula*, not only because of their higher number and bigger size, but also by allowing a stronger response to chemical stimuli [22].

According to these hypotheses, and although little is known about *L*. *delicatula* biology [12] and not much more for other planthoppers [19, 20], a possible scenario on how these antennal sensory structures might be related to some basic behaviours in *L*. *delicatula* is suggested.

During planthopper evolution, olfactory functions that were based on the antenna were transferred to the labium, particularly the short-range host plant recognition based on contact chemoreception and taste was transferred to the tip of the labium [27, 28]. In parallel, host plant long distance recognition, usually made by flagellum receptors, switched to the pedicel, which developed this sensory function via powerful sensory plate organs. Flagellum receptor reduction probably occurred independently several times in the Hemiptera but started from the apex of flagellum [39]. We suggest that in first instar planthoppers, the paired flagellum sensilla placodea (PO1) still maintain the original general and long distance host plant recognition function that is progressively lost in older instars during their heterometabolic development. However, because species dispersion and particularly host-plant recognition are essential for phytophagous planthoppers, these functions were maintained during evolution of the taxa by their transfer to the pedicel. These functions develop progressively during the nymphal instars via a progressively enriching network of diverse pedicel sensilla. It is probable that this functional shift facilitated species to specialise progressively on host plants, developing poly-, oligo- or monophagous diet preferences, assisted by related morphological changes of pedicel sensilla, particularly for sensilla placodea.

In *L*. *delicatula* the flagellar sensilla placodea PO1 may reflect such an ancestral function of less specialized, long distance host-plant recognition. Indeed, as eggs usually hatch on a suitable host plant selected by the female, first instars do not face difficulties in finding suitable feeding sites and long distance orientation is not essential at this stage [50]. In the second instar, more specialized sensilla placodea (PO2) start to develop on the antenna pedicel, initializing dispersion behaviour for the species [3]. They function in long distance recognition of *Ailanthus*, a preferred, but non-exclusive, host plant. Both the increasing attraction from second instar to adults for *Ailanthus* [3], the morphology of the nymphal antenna better dorsally oriented to explore the surrounding area seeking *Ailanthus* tree feeding sites, and they greater number in adults support this hypothesis.

*L*. *delicatula* is considered as a polyphagous species, but even if *Ailanthus* might not be an obligatory host-plant, a short feeding period on *Ailanthus* might be necessary. In adults, and particularly for females, attraction for *Ailanthus* is strong as shown by sticky trap catches on tree trunks [48]. This is probably related to the search by females for feeding and probably also oviposition sites on *Ailanthus*. The shift of a larger sensory surface to the antennal ventral faces would facilitate location of the preferred feeding and/or oviposition sites. Intraspecific competition for feeding sites between adult planthoppers and unreported occurrence of swarms (E. Smyers pers. comm.) supports this hypothesis (and suggests also that optimal, but limited, feeding sites exist on trees).

Short-range male-female communication with acoustic behaviour has not yet been reported but very probably [14] occurs in *L*. *delicatula* and this should be analysed in order to document possible premating behaviour. Also possible pheromone-driven intraspecific interactions, although yet never reported in planthoppers need to be investigated. How chemical communications and short range acoustic interact in *L*. *delicatula* need also to be further explored.

## Conclusion

As a conclusion, it is however important to note that correlations, as reported in this paper, do not necessarily imply causality or even any specific linkage. These are currently new working hypotheses that will require to be evaluated using more behavioural data in *L*. *delicatula* and other planthoppers taxa before being accepted and generalized to planthoppers. Evidences from more sophisticated approaches, starting with electrophysiological responses to volatile organic compounds selectively tested for each sensilla type will be also needed to confirm them.

## Supporting information

S1 Appendix Measurements with length (L, μm) and basal diameter (BD, μm) of antennal sensilla in the *Lycorma delictula* for all stages. (DOCX)

S2 Appendix Evolution at the different stages (N1, N2, N3, N4) and sex (M, F) of number of sensory plate organs (PO) and their estimated sensory surface (SS), for one antenna (Ant) and per antennal face—anterior (AF), dorsal (DF), posterior (PF) and ventral face (VF)—in *L*. *delicatula*. All measurements in μm and according to the following formula: Sensory Surface = S Plate + (S Lobe x nb lobes) = ((ØPO/2)^2^ x ∏) + ((2 x ((ØLob/2)^2^ x ∏)) x NbLob).
(DOCX)
