# Supplementary material for: Relating antennal sensilla diversity and possible species behaviour in the planthopper pest Lycorma delicatula (Hemiptera: Fulgoromorpha: Fulgoridae)
Source: PLoS One. 2018 Mar 27;13(3):e0194995. doi: 10.1371/journal.pone.0194995 (PMC5871016; doi:10.1371/journal.pone.0194995)
Supplement: S1 Appendix — (DOCX) [file pone.0194995.s001.docx]

**Supporting information**

**S1 Appendix. Measurements with length (L, μm) and basal diameter (BD, μm) of antennal sensilla in the *Lycorma delictula* for all stages**

| Sensilla | | Nymphal stage | | | | Adult | |
| --- | --- | --- | --- | --- | --- | --- | --- |
|  |  | **N1**  **(n=11)** | **N2**  **(n=8)** | **N3**  **(n=10)** | **N4**  **(n=10)** | **Male**  **(n=6)** | **Female**  **(n=6)** |
| Ch1 (n=33) | **n** | 9 | 4 | 7 | 6 | 2 | 3 |
|  | **L - BD** | 31.07 - 4.70 | 20.55 - 3.55 | 21.79 - 4.90 | 24.92 - 4.49 | 35.15 - 6.69 | 25.12 - 5.32 |
|  | **L/BD** | 6.6 | 5.8 | 4.5 | 5.6 | 5.3 | 4.7 |
| Ch2 (n=19) | **n** | 6 | 5 | 2 | 4 | absent | 2 |
|  | **L - BD** | 38.23 - 2.45 | 46.51 - 2.65 | 56.14 -3.11 | 67.29 - 3.39 | - | 46.83 - 2.46 |
|  | **L/BD** | 15.6 | 17.2 | 18.1 | 19.85 | - | 19 |
| Ch2L (n=5) | **n** | absent | absent | absent | 2 | 3 | / |
|  | **L - BD** | - | - | - | 76.5 - 4.2 | 76.5 - 4.2 | / |
|  | **L/BD** | - | - | - | 9.55 | 9.55 | / |
| Ch3 (n=4) | **n** | 2 | present | 2 | present | present | present |
|  | **L - BD** | 20.87 - 2.20 | / | 31.82 - 3.32 | / | / | / |
|  | **L/BD** | 9.5 | / | 9.6 | / | / | / |
| PO1 (n=25) Large/small unit | **n** | 11 | 8 | 6 | absent | absent | absent |
|  | **BD** | 70.0 / 40.0 | 34.0 / 24.0 | 22.0 - 12.0 | - | - | - |
| PO2 (n=176) | **n** | absent | 40 | 82 | 33 | 8 | 13 |
|  | **BD** | - | 39.55 | 44.79 | 51.83 | 44.96 | 52.71 |
| Ca1 (n=25) | **n** | 4 | 5 | 8 | 4 | 2 | 3 |
|  | **BD** | 13.98 | 13.86 | 15.06 | 17.57 | 14.04 | 17.70 |
| Ca2 (n=12) | **n** | absent | absent | absent | 2 | 5 | 5 |
|  | **BD** | - | - | - | 11.50 | 9.99 | 10.31 |
| BO (n=19) | **n** | 3 | 5 | 4 | 2 | 2 | 3 |
|  | **BD** | 8.24 | 5.52 | 3.57 | 3.38 | 8.53 | 9.53 |

Abbreviations: BO, Bourgoin sensory organ; BD: sensillum basal diameter; Ca1, Ca2, sensilla campaniformia subtypes; Ch1, Ch2, Ch2L, Ch3, sensilla chaetica subtypes; n, number of samples mesured; L, sensillum lenght; N1, N2, N3, N4, nymhal stages; PO1, PO2, plate organ subtypes; -, not applicable; /, present but not mesured; n, number of samples mesured.
