# Supplementary material for: Relating antennal sensilla diversity and possible species behaviour in the planthopper pest Lycorma delicatula (Hemiptera: Fulgoromorpha: Fulgoridae)
Source: PLoS One. 2018 Mar 27;13(3):e0194995. doi: 10.1371/journal.pone.0194995 (PMC5871016; doi:10.1371/journal.pone.0194995)
Supplement: S2 Appendix — All measurements in μm and according to the following formula: Sensory Surface = S Plate + (S Lobe x nb lobes) = ((ØPO/2)2 x ∏) + ((2 x ((ØLob/2)2 x ∏)) x NbLob). (DOCX) [file pone.0194995.s002.docx]

**S2 Appendix.** **Evolution at the different stages (N1, N2, N3, N4) and sex (M, F) of number of sensory plate organs (PO) and their estimated sensory surface (SS), for one antenna (Ant) and per antennal face - anterior (AF), dorsal (DF), posterior (PF) and ventral face (VF) - in *L. delicatula*. All measurements in μm and according to the following formula: Sensory Surface = S Plate + (S Lobe x nb lobes) = ((ØPO/2)^2^ x ∏) + ((2 x ((ØLob/2)^2^ x ∏)) x NbLob).**

| **Stage** | | **N1** | | **N2** | | | **N3** | | | **N4** | **M** | **F** |
| --- | --- | --- | --- | --- | --- | --- | --- | --- | --- | --- | --- | --- |
| **PO** | | **LPO1** | **SPO1** | **LPO1** | **SPO1** | **PO2** | **LPO1** | **SPO1** | **PO2** | **PO2** | **PO2** | **PO2** |
| **Ø PO (μm)** | | 70 | 40 | 34 | 24 | 39.55 | 22 | 12 | 44.79 | 51.83 | 44.96 | 52.71 |
| **S PO (S1, μm^2^)** | | 3 848.5 | 1 256.6 | 907.9 | 452.4 | 1 228.5 | 380 | 113.1 | 1 575.6 | 2 109.9 | 1587.6 | 2182.1 |
| **nb Lob (nL)** | | 68 | 27 | 27 | 16 | 22 | 14 | 7 | 28 | 37 | 31 | 36 |
| **Ø Lob (μm)** | | 10 | 10 | 5 | 5 | 8 | 5 | 5 | 10 | 10 | 10 | 10 |
| **S Lob x nL x 2 (S2, μm^2^)** | | 10681.4 | 4241.2 | 1060 | 628.4 | 2211.6 | 549.8 | 274.8 | 4398.2 | 5812 | 4869.4 | 5654.8 |
| **S PO (S1+S2) (μm^2^)** | | 14529.9 | 5497.8 | 1 969.9 | 1080.8 | 3440.1 | 929.8 | 387.9 | 5973.8 | 7921.9 | 6457 | 7836.9 |
| **DF** | **n** | 0 | 0 | 0 | 0 | 5 | 0 | 0 | 16 | 32 | 56 | 83 |
|  | **DF TSS (μm^2^)** | - | - | - | - | 17200.5 | - | - | 95580.8 | 253500.8 | 361592 | 650462.7 |
| **AF** | **n** | 1 | 0 | 1 | 0 | 5 | 1 | 0 | 12 | 26 | 63 | 109 |
|  | **SS (μm^2^)** | 14529.9 | - | 1 969.9 | - | 17200.5 | 929.8 | - | 71685.6 | - | - | - |
|  | **AF TSS (μm^2^)** | 14529.9 | | 19170.4 | | | 72615.4 | | | 205969.4 | 406791 | 854222.1 |
| **VF** | **n** | 0 | 0 | 0 | 0 | 4 | 0 | 0 | 11 | 22 | 88 | 138 |
|  | **VF TSS (μm^2^)** | - | - | - | - | 13760.4 | - | - | 65711.8 | 174281.8 | 568216 | 1081492.2 |
| **PF** | **n** | 0 | 1 | 0 | 1 | 6 | 0 | 1 | 13 | 26 | 65 | 97 |
|  | **SS (μm^2^)** | - | 5497.8 | - | 1080.8 | 20640.6 | - | 387.9 | 77659.4 | - | - | - |
|  | **PF TSS (μm^2^)** | 5497.8 | | 16 143.4 | | | 78047.3 | | | 205969.4 | 419705 | 760179.3 |
| **Total** | **n** | 1 | 1 | 1 | 1 | 14 | 1 | 1 | 44 | 84 | 210 | 320 |
|  | **SS (μm^2^)** | 14529.9 | 5497.8 | 1 969.9 | 1080.8 | 48 161.4 | 929.8 | 387.9 | 262847.2 | - | - | - |
|  | **Ant TSS (μm^2^)** | 20027.7 | | 51212.1 | | | 264164.9 | | | 665439.6 | 1355970 | 2507808 |
| Increasing factor between consecutive stages /  with 1st instar | | -  - | | x2.6  x2.6 | | | x5.2  x13.2 | | | x2.5  x33.2 | x2  x67.7 | x3.8  x125.2 |

Abbreviations: Ø PO, PO diameter;; nb Lob (nL), number of lobes per PO; Ø Lob, Lobe diameter; S Lob x nL x 2 (S2), calculated surface of all lobes for one PO; S PO (S1), sensory surface for one PO disc; TSS, total sensory surface.
